# iSeqQC: a tool for expression-based quality control in RNA sequencing

**DOI:** 10.1186/s12859-020-3399-8

**Published:** 2020-02-13

**Authors:** Gaurav Kumar, Adam Ertel, George Feldman, Joan Kupper, Paolo Fortina

**Affiliations:** 1 Cancer Genomics and Bioinformatics Laboratory, Sidney Kimmel Cancer Center, Department of Cancer Biology, BLSB 1009, Thomas Jefferson University, 233 South 10th Street, Philadelphia, PA-19107 USA; 2 Department of Orthopedic Research, Thomas Jefferson University, Philadelphia, PA USA

**Keywords:** RNA sequencing quality control, Count based QC, Expression-based QC, RNA seq QC tool

## Abstract

**Background:**

Quality Control in any high-throughput sequencing technology is a critical step, which if overlooked can compromise an experiment and the resulting conclusions. A number of methods exist to identify biases during sequencing or alignment, yet not many tools exist to interpret biases due to outliers.

**Results:**

Hence, we developed iSeqQC, an expression-based QC tool that detects outliers either produced due to variable laboratory conditions or due to dissimilarity within a phenotypic group. iSeqQC implements various statistical approaches including unsupervised clustering, agglomerative hierarchical clustering and correlation coefficients to provide insight into outliers. It can be utilized through command-line (Github: https://github.com/gkumar09/iSeqQC) or web-interface (http://cancerwebpa.jefferson.edu/iSeqQC). A local shiny installation can also be obtained from github (https://github.com/gkumar09/iSeqQC).

**Conclusion:**

iSeqQC is a fast, light-weight, expression-based QC tool that detects outliers by implementing various statistical approaches.

## Background

High-throughput experiments are complex and prone to numerous biases during sample preparation, library preparation and sequencing. Therefore, Quality Control (QC) is critical and if overlooked, can compromise the data. To reduce false discoveries from any quantitative sequencing experiment such as RNA-seq, miRNA-seq and ATAC-seq, QC can be categorized in three different phases. In phase one, quality of raw read sequences is analyzed to detect bad quality bases. This is mainly performed on raw FASTQ files using tools including FastQC [1], FASTX-Toolkit [2], NGS QC Toolkit [3] and PrinSeq [4]. In phase two, mapping quality, read count distribution, mean insert size distribution, mean depth distribution, GC-content, base quality and capture efficiency are observed on aligned BAM files to detect sample biases occurring during library preparation. This is mainly done using tools like RseQC [5], RNA-SeQC [6], QC3 [7], QoRTs [8], and Qualimap [9]. In phase three, there are yet no defined rules to perform QC on expression data. Presently, sample heterogeneity, outliers and any cross-sample contamination are detected using various statistical approaches such as correlations and dimensional reductions. There are a few tools available including EDASeq [10], NOISeq [11] and DEGreport [12], but they require bioinformatics or programming savviness for implementation. Hence, no simplified tool is available to provide QC on expression data in a comprehensive manner for the detection of outliers in any sequencing experiment.

Here, we present iSeqQC- a simple expression-based quality control tool to detect outliers either produced due to variable laboratory conditions, reagent lots, personnel differences, different experiment times, or merely due to dissimilarity within a phenotypic group. Very straight-forward to use, iSeqQC uses a raw read count matrix or normalized transcript expression data to produce QC metrics in the form of graphical plots defining relationships of all samples.

## Implementation

### iSeqQC algorithm

iSeqQC provides comprehensive information to identify any outliers in the sequencing experiment due to any technical biases. Developed in R, it can be utilized through a Shiny Server web interface (http://cancerwebpa.jefferson.edu/iSeqQC), command-line or the source package can be downloaded from https://github.com/gkumar09/iSeqQC.

iSeqQC requires two tab-delimited text files to execute: 1) a sample phenotype file with information on sample names, phenotypes and/or any confounding factors, if available; 2) a count matrix (either raw or normalized) with gene id or symbol from any read summarization tool such as RSEM [13], HTseq [14], featureCounts [15], Kallisto [16], Salmon [17] and so on. Using information from sample phenotype file, iSeqQC first matches the sample names to the count matrix, then implements the following statistical approaches to provide comprehensive QC metrics:

1. Summary statistics and counts distribution: it uses the expression matrix to provide basic descriptive summary statistics and a normally distributed expression matrix to provide counts distribution per sample.
2. Mapped reads density: For human or mouse organism, it uses Transcripts Per Million (TPM) normalization on raw expression matrix using the following formula, density of mapped reads is estimated for each sample

$$ \mathrm{TPM}=t\Big(t\left(\frac{c}{l}\right)\times {10}^6/ colSums\left(\frac{c}{l}\right) $$).

where c is the total number of reads mapped to a gene and l is the length of a gene. For other organisms, it uses DESeq2 variance stabilized normalization.

3. Housekeeping gene expression: Expression profile (log2 scale) of two housekeeping genes, GAPDH and ACTB for all samples.
4. Principal Component analysis (PCA)- Normalized: After z-transforming the expression matrix so that each row has a mean of 0 and a variance of 1, PCA, a dimensionality reduction algorithm is implemented to linear transform the data and observe the variance between samples. Z-score normalization is performed using
  $$ \mathrm{z}=\frac{\left(x-\upmu \right)}{\upsigma} $$

where μ is mean and σ is variance.

Principal component analysis (PCA)- Un- normalized: To observe the variance between samples without any standardization, PCA on raw expression matrix without any normalization is also implemented.

5. Multiple factor analysis: In a sequencing experiment, external factors that are not of biological interest can affect the expression of individual samples [18]. To observe the contribution of multiple co-variates that can contribute to the active variables to define the distance between samples multifactorial PCA was implemented. It implements ‘MFA’ function from FactoMineR package [19] in R/Bioconductor.
6. Hierarchical relationship: Measuring the distance of similarity between samples, agglomerative hierarchical clustering using Euclidean distance method is also implemented on the normally distributed expression matrix.
7. Correlation: For correlation association, Pearson (quantity-based) and Spearman (rank-based) correlation using Ward’s method is implemented on the expression matrix.
8. GC-bias: To assess if the bias is due to any error in library preparation step, the Locally Weighted Scatterplot Smoothing (LOWESS) fit of gene-count for each sample is plotted against GC-content. To obtain the obtain gene length and GC content for both human (GRCh38) and mouse (GRCm38), ‘getGeneLengthAndGCContent’ algorithm from EDAseq [10] was used.
9. Expression plot: Expression profile of any gene of interest.

After successfully executed, iSeqQC provides QC metrics in the form of a table and several graphical plots. First, it uses expression data to provide descriptive statistics, output in a form of a ‘summary statistics’ table. For each sample, it provides number of detected genes, mean expression, standard deviation, median expression, minimum expression, maximum expression, range of expression, skewness (symmetry of expression distribution), kurtosis (tails of distribution), library size and number of expressed genes (genes with greater than 0 reads). Next, the expression data is displayed as a ‘count-distribution’ box-plot to provide overall distribution of the expression of each sample with minimum, maximum and median expression. Further, the expression data is normalized (TPM for human or mouse and DESeq2 variance stabilized normalization for other organisms) and density distribution of mapped reads is provided in a form of ‘mapped read density’ plot to observe any sample with no or low expressing reads. Due to stable expression of housekeeping genes, they are often used in sequencing experiments to normalize mRNA levels between different samples. iSeqQC uses GAPDH and ACTB expression data to detect whether samples show high expression of these two genes. All this information is used as a first sign to detect any outlier and could be further investigated for any possible biasness.

After implementing basic statistics algorithms, iSeqQC implements various dimensionality reduction approaches to extract any technical bias in the sequencing experiment. Here, it first z-score normalizes the expression data and implements PCA unsupervised clustering method to identify the principal directions or variations called as components. The first two principal components which mainly are the highest source of variance are then displayed as a plot. This plot further segregates the samples based on their phenotype (data obtained from sample phenotype sheet). In some cases, normalizing the data can mask systematic bias, hence, iSeqQC also implements PCA on un-normalized data. To test the effect of external factors that are not of biological interest, but are possible sources of systematic bias, iSeqQC implements multifactor PCA. Here, multiple variables are weighted and assigned a weight equal to the inverse of the first eigenvalue of the analysis. Further, hierarchical clustering is implemented to provide the distance of similarity between replicates in a specific phenotypic group. Using the agglomerative method, it assigns each sample to its own cluster and then computes distance between each cluster and joins the two most similar clusters together. Next, iSeqQC utilizes correlation coefficients to detect the strength and direction of the relationship between the samples. It uses Pearson correlation, which evaluates the linear relationship between the samples and Spearman correlation, which is a rank-based method that can range from − 1 to + 1. The direction of the relationship is indicated by the value of the coefficient; samples with a close relationship tend to be in the positive range and vice versa. Next, relationship of read counts and GC-content for each sample is plotted to illustrate any bias in sequencing libraries. Output in the form of a plot, all this comprehensive information cumulatively provides sufficient indication of any outlier sample or cross-sample contamination.

Additionally, iSeqQC provides an option to plot expression levels of any gene of interest for all samples in a sequencing experiment.

### Sample collection

The blood samples from Depuytrens affected patients and controls were collected and stored in RNAlater (ThermoFischer Scientific, MA, USA, catalogue no.-AM7020) at -80 °C. The samples were collected under the Institutional Review Board (IRB) approval #17D.510 of Thomas Jefferson University Hospital and informed consent was obtained from each participant. Under sterile conditions, total RNA was extracted using the Qiagen miRNeasy mini-kit (Qiagen, MD, USA, catalogue no.-217,004).

### RNA extraction, library preparation and sequencing

4 ng of total RNA was used to prepare libraries using the Takara Bio SMARTer Stranded Total RNA-Seq Kit (Takara Bio, CA, USA, catalogue no.- 634,837) following manufacturer’s protocol. The final libraries were sequenced on NextSeq 500 using 75 bp paired-end chemistry.

### Alignment

Raw FASTQ sequencing reads were mapped against the reference genome of *Homo sapiens* Ensembl version GRCh38 utilizing further information from the gene transfer format (.gtf) annotation from GENCODE version GRCh38.p12 using STAR aligner [20] utilized through RSEM [13]. Total read counts, and normalized Transcripts Per Million (TPM) were obtained using RSEM’s calculate-expression function.

### Sequencing and library QC

Sequencing QC to obtain any read errors, poor quality reads and primer or adapter contamination was observed using FastQC [1]. Inconsistencies in sample and library preparation was observed using QC3 [7], QoRTs [8], and RSeqQC [5].

### Differential expression analysis

Differential gene expression was performed using diseased and control samples using the DESeq2 [21] package in R/Bioconductor. Genes were considered differentially expressed (DE) if they had adjusted *p* value ≤0.05 and absolute fold change ≥2. All plots were constructed using R/Bioconductor.

### Publicly available datasets

To demonstrate the utility of iSeqQC, we also used previously studied datasets. We obtained sequence read archive FASTQ files of the Bottomly et al. [22] mouse RNA-seq dataset (accession number [SRP004777]) from two different strains mice (B6 and D2). Reads were aligned to *Mus musculus* (GRCm38) using STAR aligner implemented through RSEM. Total read counts were obtained using RSEM. This dataset was also used by Love et al. [21] to test DESeq2 performance. Similarly, raw FASTQ reads for yeast dataset (accession number [SRA048710]) by Risso et al. [10], were aligned to *Saccharomyces cerevisiae* (R64–1-1) to obtain raw expression matrix. Additionally, raw count matrix of ENCODE dataset of human B-cells [23] used by Tarazona et al. [11] was obtained from the NOISeq web-page (http://bioinfo.cipf.es/noiseq/doku.php).

## Results

To demonstrate the importance of expression QC and performance of iSeqQC, we first utilized RNA-seq samples sequenced in our laboratory to study Dupuytren’s disease.

Following our laboratory standard protocols, all samples were tested to access RNA Integrity Number (RIN) and were within range of the requirements of the library preparation kit (> 2). The disease samples had a RIN score between 4 and 5 and control samples were between 2 and 3.

**Fig. 1:**
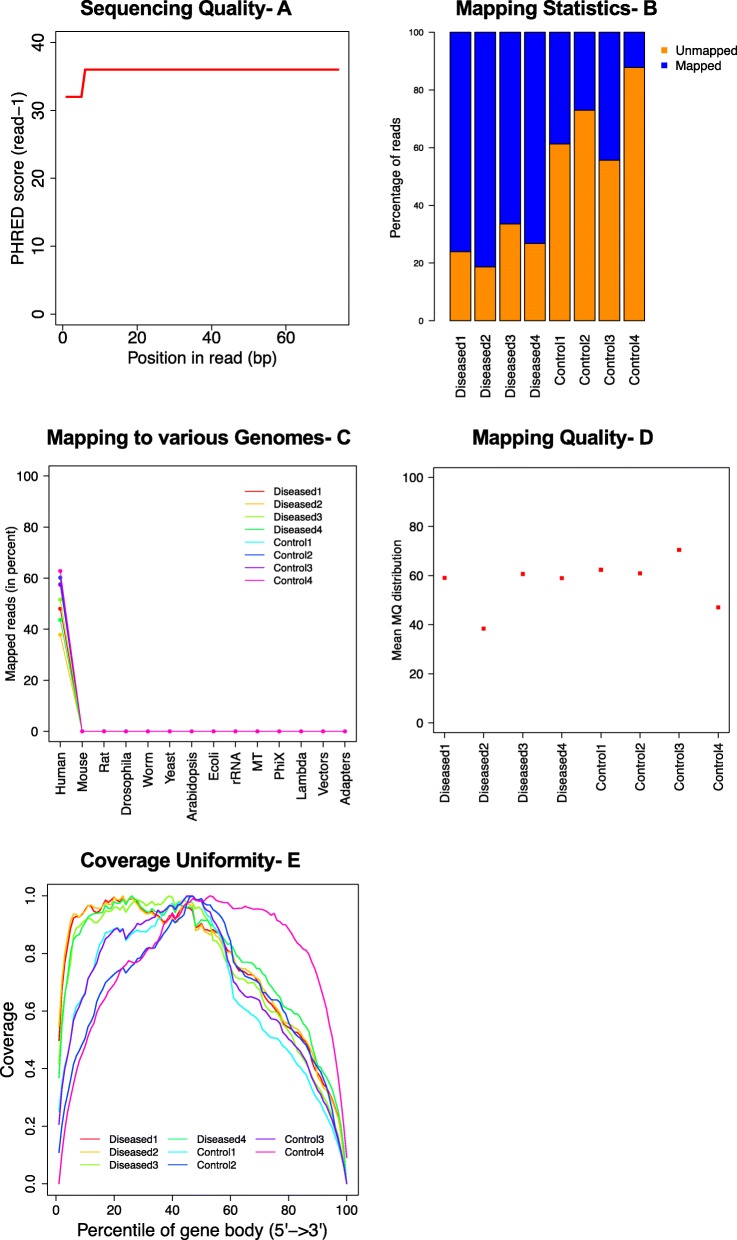
Quality control metrics using existing tools. **a** Per base sequencing quality averaged for all diseased and control samples; **b**) Mapping statistics showing percentage of mapped versus unmapped reads in diseased and control samples; **c**) Percentage of reads mapped uniquely to human genome demonstrating no contamination in the libraries; **d**) Average mapping quality showing no outlier; **e**) Coverage uniformity over gene body for all samples showing no outlier

Samples were sequenced and resultant FASTQ files were examined for sequencing errors using FastQC in phase one of QC. Here, for all samples, per base sequence quality for all bases at each position was observed to be > 30, demonstrating a base call accuracy > 99.9% (Fig. 1a). Additional metrics such as per base sequence and GC content also were observed to be good quality.

In phase two, QC was observed on the aligned BAM files using QC3 and RSeqQC. QC3 was used to observe mapping statistics, where all diseased samples had > 70% reads mapped to the reference genome showing high quality RNA samples. However, control samples had an overall low mapping percentage (control1, 2, 3: ~ 40% and control4: ~ 20%) as shown in Fig. 1b. With the suspicion of DNA or any other contamination, we used FastQ Screen to further investigate the low mapping of control samples. A high proportion of mapped reads were mapping to only the human genome (Fig. 1c). Next, RSeqQC was used to observe average mapping quality, where diseased2 and control4 were observed to have a low but acceptable quality as shown in Fig. 1d. Additionally, the coverage over gene body analysis also showed an acceptable coverage uniformity over gene body in all samples (Fig. 1e). These existing sequencing and library QC tools were inconclusive to detect any outliers in the study.

**Fig. 2:**
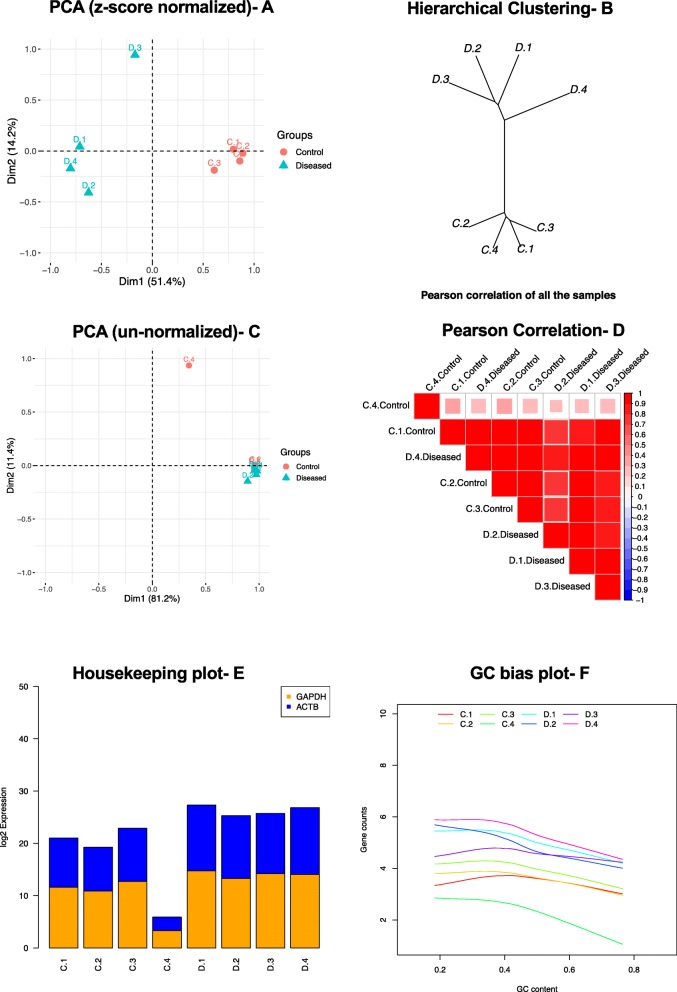
Quality control metrics produced by iSeqQC. **a**) Unsupervised PCA clustering (z-scored normalized) showing tight cluster of samples within the phenotype; **b**) Hierarchical relationship assigning each sample to its own phenotypic cluster; **c**) Unsupervised PCA clustering (un-normalized) showing control4 to be phenotypically different; **d**) Pearson correlation showing relationships between samples among biological replicates; **e**) Normalized expression of housekeeping genes (GAPDH and beta-actin) among different samples showing low expression of control4 sample; **f**) GC bias plot showing control4 with lower gene-counts relative to GC content

Next, in phase three, QC was observed on expression data using iSeqQC, which generates a summary table and 10 different plots to infer QC. Upon investigating the Principal Component Analysis (PCA) clustering (z-scored normalized) (Fig. 2a) and hierarchical clustering (Fig. 2b) from iSeqQC output, we observed tight clustering and correlation of each sample in its phenotypic group, hence no biases. However, the PCA clustering on un-normalized data (Fig. 2c) and Pearson correlation (Fig. 2d) showed control4 dissimilar from rest of the samples. Furthermore, the ‘housekeeping gene’ plot showed an overall low expression of ACTB and GAPDH in control4 sample when compared to other samples tested (Fig. 2e). Similarly, the ‘summary statistics’ table also showed low expression of all detected genes in control4 (Table 1). These QC results by iSeqQC indicated that due to its low-expression profile control4 sample could be considered as an outlier. Further, examining the ‘GC-bias’ plot (Fig. 2f) showed control4 sample’s GC-content profile to be lower when compared to other samples, inferring library-preparation could be the source of this bias. Since, there was no confounding factor in our dataset, iSeqQC did not compute the multifactor PCA. Remaining output plots from iSeqQC are provided as supplement data (Additional file 1 **iSeqQC_outputs**). A comparison of existing QC tools and iSeqQC is provided in Table 2 indicating its importance in overall QC in expression-based sequencing experiments.

**Table 1:** Summary statistics of control and diseased samples showing overall low expression of control 4 sample (iSeqQC output)

Samples names	Detected Genes	Mean	SD	Median	Min	Max	Range	Skew	Kurtosis	Library Size	Expressed Genes
C.1	31,963	57.21	527.06	13.34	0	47,932	47,932	56.42	2.95	1,550,389	30,495
C.2	31,963	48.51	404.42	16.31	0	46,324	46,324	81.98	2.26	2,779,530	29,821
C.3	31,963	86.96	805.74	14.83	0	87,803	87,803	73.67	4.51	336,318	28,946
C.4	31,963	10.52	15.42	7.41	0	829	829	11.57	0.09	11,606,701	25,414
D.1	31,963	363.13	5348.91	22.24	0	633,897	633,897	81.32	29.92	9,982,421	24,625
D.2	31,963	312.31	8118.42	19.27	0	1,223,093	1,223,093	117.76	45.41	6,433,195	27,140
D.3	31,963	201.27	2433.88	20.76	0	289,596	289,596	71.74	13.61	14,027,954	24,074
D.4	31,963	438.88	5840.56	28.17	0	685,534	685,534	78.72	32.67	1,550,389	30,495

**Table 2:** Features and capabilities of iSeqQC compared with other tools

Metrics	iSeqQC	QoRTs	QC3	RSeqQC	RNA-SeQC	EDASeq	NOISeq
Summary expression	Yes	No	No	No	No	Yes	No
Dimensional reduction	Yes	No	No	No	No	No	Yes
Correlations	Yes	Yes	Yes	Yes	Yes	No	No
Housekeeping genes expression	Yes	No	No	No	No	No	No
Generate Plots	Yes	Yes	No	No	No	No	No

**Fig. 3:**
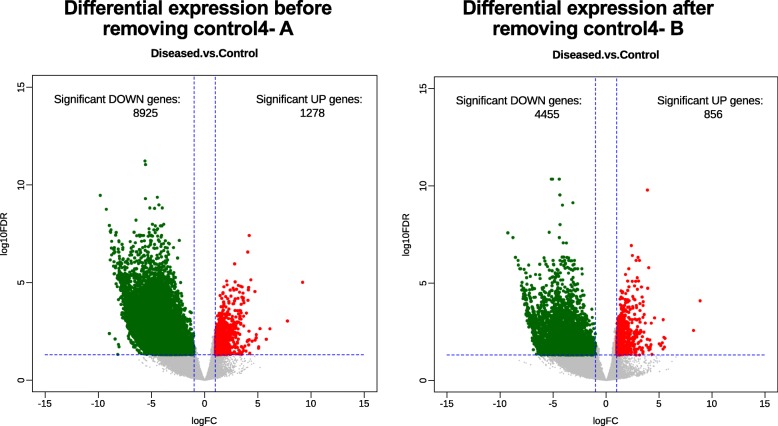
Change in number of differentially expressed genes before and after removal of outlier from differential expression analysis demonstrating the impact of an outlier in differential expression

Even though, iSeqQC flagged control4 to be an outlier, we decided to include it in further analysis for demonstration purposes. We performed differential expression analysis to obtain genes that are modulated in disease (absolute fold change> 2 and adjusted *p* value< 0.05) when compared to control. Here, we obtained 10,203 differentially expressed genes (DEGs), where 1278 were significantly up-regulated and 8925 were significantly down-regulated (Fig. 3a). To access the impact of the outlier, we removed control4 sample (by changing sample phenotype file as shown in the workflow in Additional file 2- **iSeqQCworkflow**) from the differential expression analysis and observed only 5311 DEGs, where 856 genes were significantly up-regulated and 4455 were significantly down-regulated (Fig. 3b). To observe the impact of outliers on biological interpretation, we examined a change in the expression of any gene with or without removing control4. Here, we found no drastic change in the expression of differentially expressed genes (common between DEGs with or without control4) if control4 was kept. This shows that in this particular case, when included in the differential expression analysis, an outlier did not change the level of gene expression but only increased the noise in the data.

Next, we tested the performance of iSeqQC on previously published datasets. In mouse dataset by Bottomly et al. [22], we observed variation among biological replicates of B6 and D2 strains mice. We also found many samples to be low-expressed (Additional file 3- **PublicDatasetResults Fig. A**). As reported originally, iSeqQC also didn’t detect any bias in the GC-content metrics (Additional file 3- **PublicDatasetResults-Fig. B**). These results by iSeqQC are in accordance with previous findings by Bottomly et al. [22]. In the Yeast dataset by Risso et al. [10], there were 11 samples of *Saccharomyces cerevisiae* grown in 3 different growth conditions: standard YP Glucose (YPD), Delft Glucose (Delft) and YP Glycerol (YP-Gly) with different library preparation methods and protocols. All the samples were sequenced on different flow cells. Z-score normalized PCA from iSeqQC showed Delft group to be tightly clustered, 2 out of 3 samples from YP-GLY were properly clustered. However, the majority of samples from YPD were dissimilar (Additional file 3- **PublicDatasetResults- Fig. C**). Upon observing the multifactor PCA, this variation could be due to significant technical variation during the experiment and library protocol/preparation. Here, iSeqQC showed library protocol method to be the major source of variation, following the use of different flow cells and library preparation (Additional file 3- **PublicDatasetResults- Fig. D**). These results are in accordance with findings by Risso et al. [10]. Finally, in the ENCODE dataset used by Tarazona et al. [11], two different RNA extracting protocols (PolyA+ extraction (Pap) and PolyA- extraction (Pam) method) were used to sequence human B-cells (CD20-) and monocytes (CD14+). Here, we observed technical variability (different RNA extracting protocols) heavily impacting the biological variability. Although, we observed all samples to be at sufficient sequencing depth, we found phenotypically different samples treated with same RNA extraction methods to be clustering well (Additional file 3- **PublicDatasetResults- Fig. E**), pointing technical bias in the dataset (Additional file 3- **PublicDatasetResults- Fig. F**). The results achieved by iSeqQC are in accordance with findings by Tarazona et al. [11].

## Discussion

Due to the complexity of high-throughput sequencing experiments, several phases of QC are required to identify any bias in the data. iSeqQC was designed to obtain comprehensive information on sample heterogeneity to detect outliers or cross-sample contamination in an expression-based sequencing experiment by implementing various statistical approaches including descriptive and dimensional reduction algorithms.

In our dataset, iSeqQC was successful in identifying an outlier that was missed by existing sequencing and library QC tools. It indicated control4 to be an outlier due to its lower expression as indicated by summary statistics, housekeeping gene expression and PCA on unnormalized metrics. Upon assessing the GC-content metrics, we believed the bias could be due to library preparation step in control4 sample. We would like to note here that upon initial look at the PCA-normalized plot, one could flag diseased3 to be an outlier. However, when metrics for this sample are evaluated as a whole, they are representative of the phenotypic group and placement on the PCA plot can be attributed to biological variation.

Data generated in our laboratory was sufficient to assess the utility of iSeqQC, but we also benchmark our tool using 3 different publicly available datasets that were tested by others previously. The well-characterized technical variance in these datasets offered high value in demonstrating the consistent performance of iSeqQC in a variety of scenarios. As expected, the results provided by iSeqQC were in accordance with the results previously reported for these datasets.

At present, there are no defined rules to perform QC on expression matrices for the detection of outliers in any sequencing experiment. As shown in the results, existing tools and algorithms may not be sufficient. iSeqQC uses ensemble of various statistical methods to provide a detailed QC metrics in the form of a table and several graphical plots to identify any outliers. Additionally, at present while performing QC using stand-alone tools researchers have to re-run several lines of codes to re-evaluate the QC after removing the outliers. With iSeqQC, it is effortless as sample can be removed from the QC analysis by simply doing a minor change in sample phenotype sheet. Also, researchers spend hours generating publishable quality QC figures such as PCA and correlations plot, however iSeqQC by-default provides high-quality publication-ready figures. We would like to note here that for count matrix with gene id as input, iSeqQC requires only Ensembl annotations. If other annotations are used, all the metrics should work except ‘housekeeping’ and ‘GC bias’. However, in that case, ‘expression plot’ can be used to obtain the expression of housekeeping genes. Also, TPM normalization in ‘mapped read density’ plot is only compatible with human and mouse data, if any other organism is used, iSeqQC uses and DESeq2 variance stabilized normalization.

While there exist several tools for assessing QC of sequencing experiments, each is limited to observe either sequencing and/or library quality. A few tools including QC3, QoRTs, RSeQC, and RNA-SeQC provide some information on outliers and cross-sample contamination but are not sufficient to provide in-depth sample qualities. QoRTs detects sample heterogeneity by analyzing read mapping, insert size distribution, cigar profile, and alignment clipping profile. RSeQC and RNA-SeQC uses Spearman and Pearson correlations to detect any outliers. QC3 is mainly focused to perform phase three QC only on Whole Exome Sequencing (WES) or Whole Genome Sequencing (WGS) data and does not include any quantitative sequencing technology such as RNA-seq. While EDASeq, NOISeq and DEGreport can utilize expression matrix as an input, they are mainly restricted to GC content, feature biotype and PCA at a basic level to explore the bias. Additionally, all these tools either require high-end computational resources or computational savviness to operate. As shown in the results, iSeqQC is simple, light-weight and accessible, yet powerful, approach to perform QC on expression-based sequencing technology,

We acknowledge that due to the complexity of wet-lab protocols in sequencing technology, there are certain biasness that can evade any standardized QC approach. Implementing statistical approaches gives only an idea of overall sample heterogeneity and is not sufficient to remove the samples from study. Use of additional methods such as Real time- Polymerase Chain Reaction (RT-PCR) is recommended to validate the findings.

## Conclusions

iSeqQC is a simple, fast, light-weight, expression-based QC tool that detects outliers by implementing various statistical approaches. Implemented through web-interface and command-line interface, it generates high-quality publication-ready QC metrics for cross-comparison of samples.

## Availability and requirements

**Project name:** iSeqQC.

**Project home page:** http://cancerwebpa.jefferson.edu/iSeqQC

https://github.com/gkumar09/iSeqQC

**Operating system(s):** Not Applicable.

**Programming language:** R.

**Other requirements:** Web browsers equal or higher Safari v-12.1, Chrome v-79.0, Firefox v-72.2.

**License:** MIT.

**Any restrictions to use by non-academics:** None.

## Supplementary information

**Additional file 1.** iSeqQC outputs. Remaining iSeqQC outputs (not included in Fig. 2). A) Counts distribution profile; B) Mapped read density profile; C) Spearman correlation showing relationships between samples among biological replicates.

**Additional file 2.** iSeqQCworkflow. Workflow describing the steps to be followed to perform QC using iSeqQC.

**Additional file 3.** Public Dataset Results. Quality control metrics produced by iSeqQC from other datasets. A) Counts distribution plot showing several low-expressed samples on Bottomly dataset; B) GC-bias plot showing no GC-content bias in any samples on Bottomly dataset; C) Unsupervised PCA clustering (un-normalized) showing variation in several samples in Risso dataset; D) Multifactor PCA showing library protocol method and different flow cell to be the major source of the variation; E) Unsupervised PCA clustering (un-normalized) showing samples clustered based on RNA extraction method in Tarazona dataset; F) Multifactor PCA showing RNA-extraction method to be the major source of variation.

## Data Availability

All the source codes and RNA-seq samples (under example directory) and dataset are available through iSeqQC webpage and github repository: https://github.com/gkumar09/iSeqQC.

## Abbreviations

DE: Differentially expressed
DEG: Differentially Expressed Genes
GTF: Gene Transfer Format
IRB: Institutional Review Board
Pam: PolyA- extraction
Pap: PolyA+ extraction
PCA: Principal Component Analysis
QC: Quality Control
RIN: RNA Integrity Number
RT-PCR: Real time- Polymerase Chain Reaction
TPM: Transcripts Per Million
WES: Whole Exome Sequencing
WGS: Whole Genome Sequencing
YPD: YP Glucose
YP-Gly: YP Glycerol
