# Supplementary figures and images for: iSeqQC: a tool for expression-based quality control in RNA sequencing
Source: BMC Bioinformatics. 2020 Feb 13;21:56. doi: 10.1186/s12859-020-3399-8 (PMC7020508; doi:10.1186/s12859-020-3399-8)

iSeqQC outputs

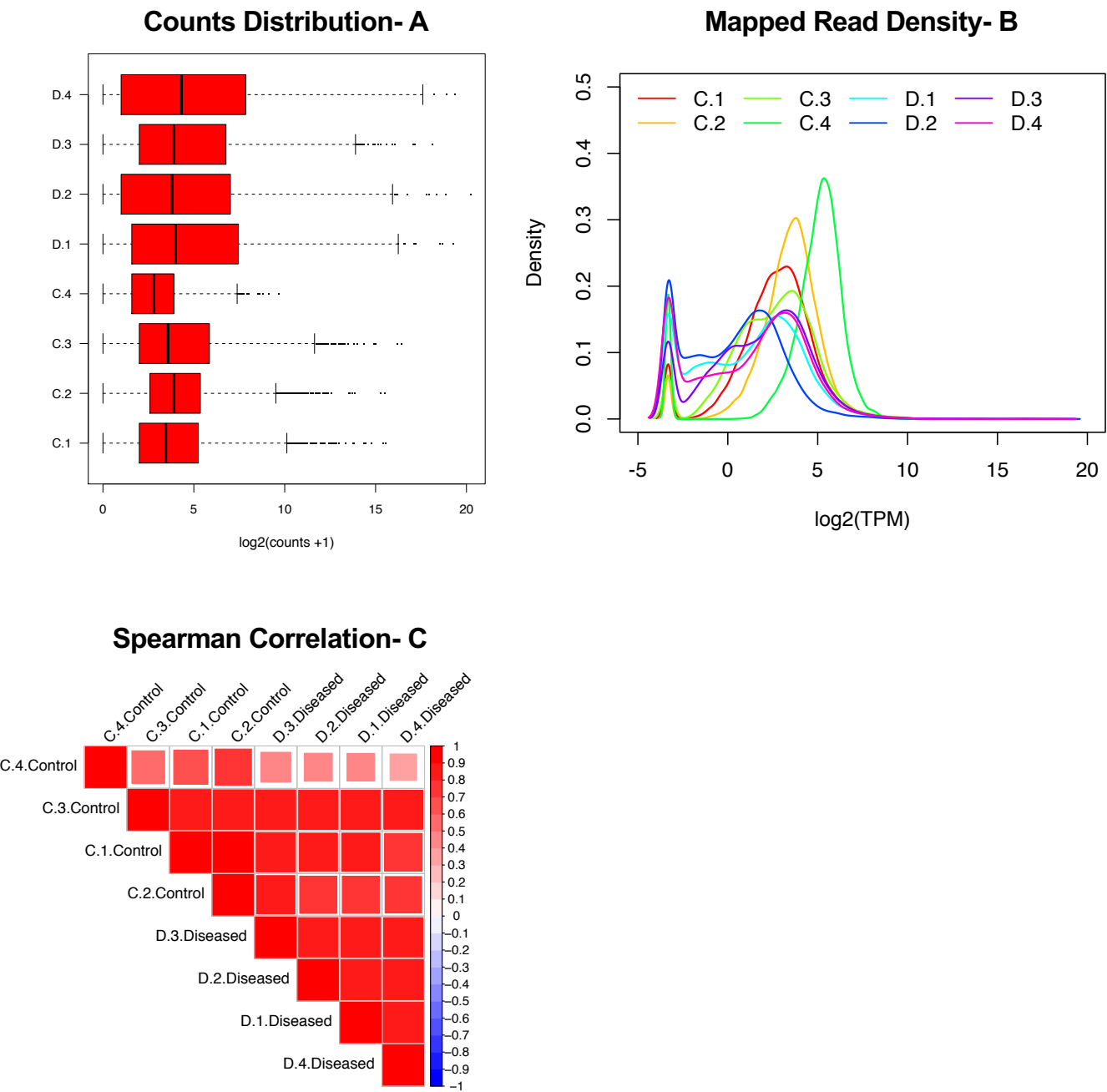

Fig. 1

Supplement: Supplementary file 1 — Additional file 1. iSeqQC outputs. Remaining iSeqQC outputs (not included in Fig. 2). A) Counts distribution profile; B) Mapped read density profile; C) Spearman correlation showing relationships between samples among biological replicates. [file 12859_2020_3399_MOESM1_ESM.pdf]

**iSeqQC workflow**

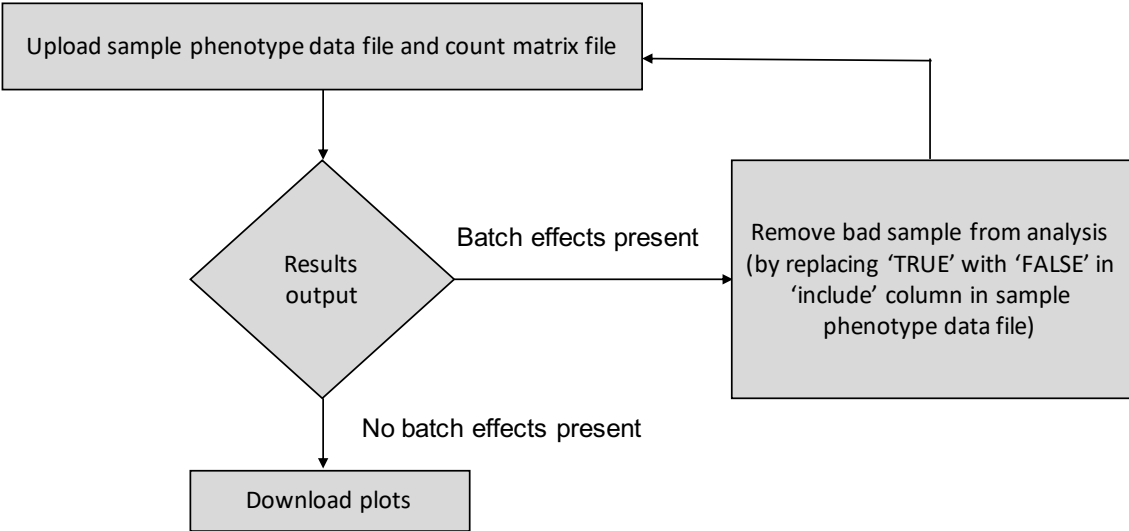

Fig. 2

Supplement: Supplementary file 2 — Additional file 2. iSeqQCworkflow. Workflow describing the steps to be followed to perform QC using iSeqQC. [file 12859_2020_3399_MOESM2_ESM.pdf]
