# Supplementary material for: iSeqQC: a tool for expression-based quality control in RNA sequencing
Source: BMC Bioinformatics. 2020 Feb 13;21:56. doi: 10.1186/s12859-020-3399-8 (PMC7020508; doi:10.1186/s12859-020-3399-8)
Supplement: Supplementary file 3 — Additional file 3. Public Dataset Results. Quality control metrics produced by iSeqQC from other datasets. A) Counts distribution plot showing several low-expressed samples on Bottomly dataset; B) GC-bias plot showing no GC-content bias in any samples on Bottomly dataset; C) Unsupervised PCA clustering (un-normalized) showing variation in several samples in Risso dataset; D) Multifactor PCA showing library protocol method and different flow cell to be the major source of the variation; E) Unsupervised PCA clustering (un-normalized) showing samples clustered based on RNA extraction method in Tarazona dataset; F) Multifactor PCA showing RNA-extraction method to be the major source of variation. [file 12859_2020_3399_MOESM3_ESM.pdf]

Public Dataset Results

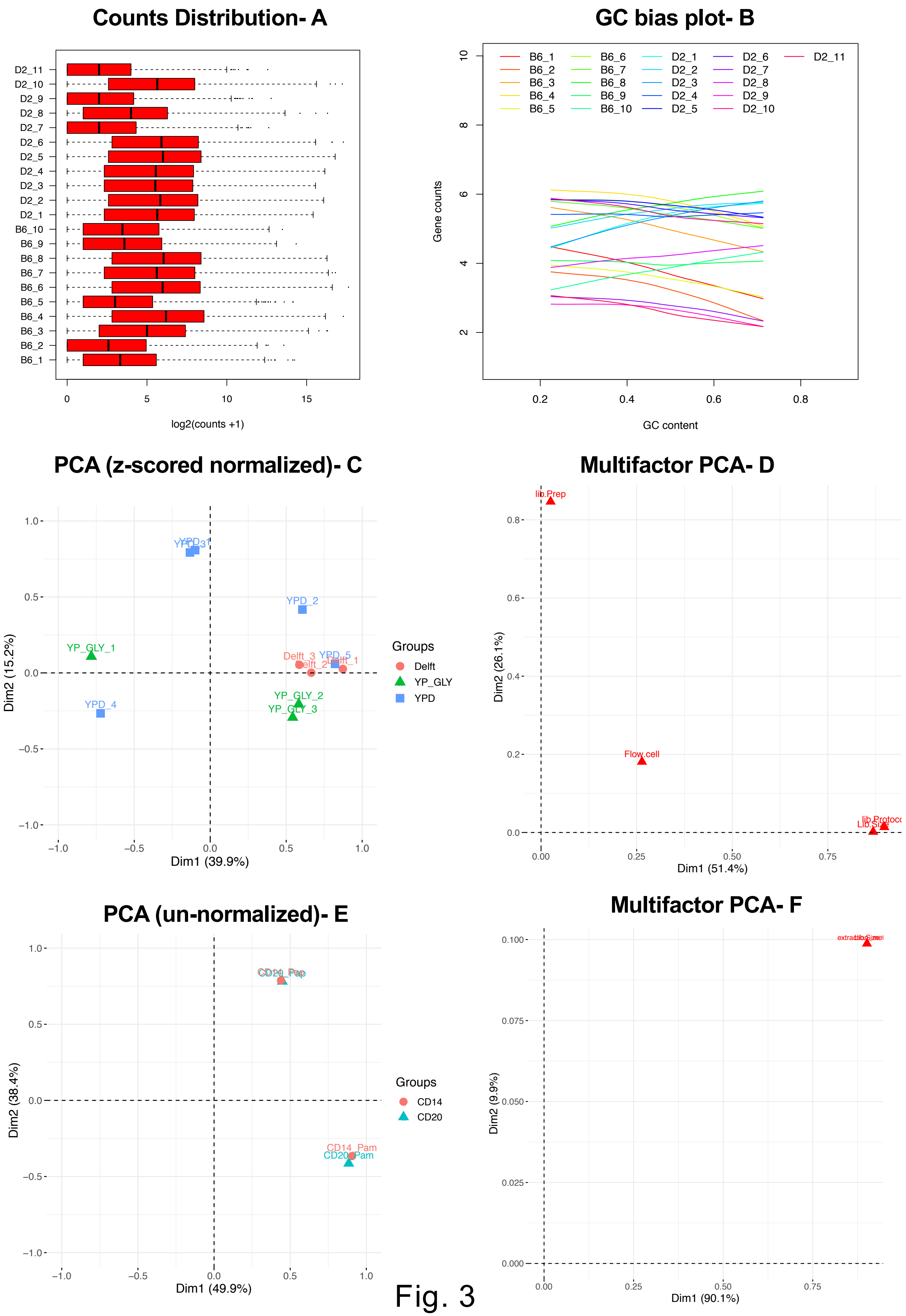

Fig. 3

iSeqQC: A Tool for Expression-Based Quality Control in RNA Sequencing
